# Noncontact Human–Machine Interface Using Complementary Information Fusion Based on MEMS and Triboelectric Sensors

**DOI:** 10.1002/advs.202201056

**Published:** 2022-05-18

**Authors:** Xianhao Le, Qiongfeng Shi, Zhongda Sun, Jin Xie, Chengkuo Lee

**Affiliations:** ^1^ Department of Electrical & Computer Engineering National University of Singapore 4 Engineering Drive 3 Singapore 117583 Singapore; ^2^ Center for Intelligent Sensors and MEMS (CISM) National University of Singapore 5 Engineering Drive 1 Singapore 117608 Singapore; ^3^ State Key Laboratory of Fluid Power and Mechatronic Systems Zhejiang University Hangzhou 310027 China; ^4^ NUS Suzhou Research Institute (NUSRI) Suzhou Industrial Park Suzhou 215123 China; ^5^ NUS Graduate School‐Integrative Sciences and Engineering Programme (ISEP) National University of Singapore Singapore 119077 Singapore

**Keywords:** bulk wave resonators, graphene oxide, human–machine interface, humidity sensors, noncontact, triboelectric sensors

## Abstract

Current noncontact human–machine interfaces (HMIs) either suffer from high power consumption, complex signal processing circuits, and algorithms, or cannot support multidimensional interaction. Here, a minimalist, low‐power, and multimodal noncontact interaction interface is realized by fusing the complementary information obtained from a microelectromechanical system (MEMS) humidity sensor and a triboelectric sensor. The humidity sensor composed of a two‐port aluminum nitride (AlN) bulk wave resonator operating in its length extensional mode and a layer of graphene oxide (GO) film with uniform and controllable thickness, possesses an ultra‐tiny form factor (200 × 400 µm^2^), high signal strength (*Q* = 1729.5), and low signal noise level (±0.31%RH), and is able to continuously and steadily interact with an approaching finger. Meanwhile, the facile triboelectric sensor made of two annular aluminum electrodes enables the interaction interface to rapidly recognize the multidirectional finger movements. By leveraging the resonant frequency changes of the humidity sensor and output voltage waveforms of the triboelectric sensor, the proposed interaction interface is successfully demonstrated as a game control interface to manipulate a car in virtual reality (VR) space and a password input interface to enter high‐security 3D passwords, indicating its great potential in diversified applications in the future Metaverse.

## Graphical abstract

By leveraging the dexterously complementary information fusion of a microelectromechanical system (MEMS) humidity sensor and a triboelectric sensor, a minimalist noncontact interaction interface with both steady and dynamic responses to finger movements is realized for high‐dimensional interactions. With the capability of detecting multimodal sensory information, the interaction interface is successfully demonstrated as a game control interface and a 3D password input interface.

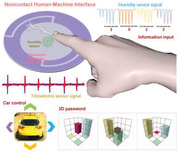

## 1 Introduction

The rise of the Metaverse concept has further increased the demand for various sensory and interactive systems to perform information exchange and mutual interactions between the real world and the virtual world, such as those that can monitor natural environmental factors, human biological and physical signals, and project such information into the virtual environment for a more intuitive, immersive, and real experience.^[^ ^1^, ^2^, ^3^ ^]^ In particular, sensory systems based control interfaces (i.e., human–machine interfaces (HMIs)) that enable humans to engage and interact with the virtual world through different motions have attracted considerable attention.^[^ ^4^, ^5^ ^]^ Thereinto, wearable HMIs are the most widely investigated domain technologies due to their unique intuitive, portable, compact, conformal, and low‐cost features (compared to conventional computer mice and keyboards).^[^ ^6^ ^]^

To realize wearable HMIs, two general directions have been widely investigated by researchers to bridge conventional electronics with soft human skins. On the one hand, the traditionally rigid electronics can be miniaturized as tiny islands and integrated on flexible substrates, with stretchable interconnects. In the past few decades, by leveraging the wafer‐level and low‐cost micromachining processes, microelectromechanical system (MEMS) sensors such as inertial and pressure sensors have achieved a huge commercial success.^[^ ^7^, ^8^, ^9^, ^10^, ^11^, ^12^ ^]^ These MEMS sensors are able to be integrated into various wearable equipment or with other wearable sensors, exhibiting good integration compatibility, low power consumption, and negligible effect on the flexible and stretchable properties of wearable HMIs.^[^ ^13^, ^14^, ^15^ ^]^ On the other hand, wearable HMIs can be directly fabricated from intrinsically flexible or stretchable materials, e.g., electronic skin,^[^ ^16^, ^17^, ^18^, ^19^ ^]^ electronic‐textile,^[^ ^20^, ^21^, ^22^, ^23^, ^24^, ^25^ ^]^ wearable patch,^[^ ^26^, ^27^, ^28^ ^]^ etc. To achieve the sensory and interactive functionality, they can be developed based on the sensing mechanisms of piezoresistive,^[^ ^29^, ^30^ ^]^ capacitive,^[^ ^31^ ^]^ piezoelectric,^[^ ^32^, ^33^ ^]^ and triboelectric effects.^[^ ^34^, ^35^, ^36^ ^]^ By contrast, the triboelectric and piezoelectric sensors are highly promising for wearable HMIs because of their self‐powered capability, especially when an enormous number of HMIs are required in the Metaverse era. Since the first invention in 2012, triboelectric nanogenerators (TENGs) based on the coupling of contact electrification and electrostatic induction and Maxwell's displacement current, have attracted worldwide interest and been investigated for diverse applications ranging from energy harvesting, self‐powered sensing, to human–machine interactions.^[^ ^37^, ^38^, ^39^ ^]^ Due to the simple configuration, low cost, wide material selection, high scalability, and good compatibility, some studies have combined the triboelectric mechanism with the piezoelectric mechanism to form hybridized HMIs, with enhanced signal multimodality and endowed multifunctionalities.^[^ ^40^, ^41^, ^42^ ^]^

In addition to wearability, noncontact sensing capability is also another desired feature for the next‐generation HMIs. Recently, we have witnessed a growing interest in wearable HMIs with noncontact attributes, especially during the current pandemic. The noncontact operation of HMIs can effectively reduce the spread of viruses and improve the user experience. Current solutions for realizing noncontact wearable HMIs include optical, radar and ultrasound sensors that can remotely detect human finger motions.^[^ ^43^, ^44^, ^45^, ^46^, ^47^, ^48^ ^]^ However, these sensors suffer from the drawbacks of high power consumption as well as complex signal processing circuits and detecting algorithms. A potential candidate to address these issues in noncontact HMIs is the triboelectric sensors with the significant advantages of self‐powered capability and facile configuration.^[^ ^49^, ^50^, ^51^ ^]^ The noncontact interaction is achieved through the electrostatic induction between triboelectric sensing elements and charged objects at a remote distance. In this regard, human fingers are ideal for such interactions since they naturally carried with charges after frequent contact with other surfaces during various daily activities.^[^ ^52^ ^]^ Nevertheless, the fly in the ointment is that the signals from triboelectric sensors are instantaneous and cannot support steady and continuous noncontact response, greatly hindering their applications in certain scenarios. Meanwhile, they normally require a large number of sensing electrodes to achieve multidirectional sensing,^[^ ^49^, ^50^ ^]^ which degrades their scalability and integration capability.

On the other hand, another promising candidate to overcome these issues is the high‐performance humidity sensors, which have the capability to keenly sense the humidity changes brought by the proximity of human fingers,^[^ ^53^, ^54^, ^55^ ^]^ especially those nanomaterials‐based humidity sensors.^[^ ^56^, ^57^ ^]^ When the water molecules diffused from the surface of an approaching finger are adsorbed by the sensing materials coated on a humidity sensor, the sensor's resistance, capacitance, or resonant frequency will change in response to the approaching of the finger.^[^ ^58^ ^]^ Meanwhile, the response of the humidity sensor is continuous during the entire process of finger movement, which provides the possibility for noncontact HMIs with steady and continuous responses.^[^ ^59^ ^]^ Amongst various humidity sensors, MEMS piezoelectric resonant humidity sensors possess the advantages of high sensitivity, high resolution, and wide sensing range with the aid of their surface humidity sensing materials.^[^ ^60^ ^]^ Moreover, their miniaturized size, low power consumption, digital frequency output, and easy integration make them suitable for use in wearable noncontact HMIs. Whereas, humidity sensor based noncontact HMIs are not ideal for instantaneous finger motion sensing such as finger sliding direction and velocity. Normally a large humidity sensor array is required, which inevitably increases the system complexity and reduces the integration compatibility with other wearable devices.^[^ ^61^ ^]^ In this regard, multimodality sensor fusion could be a more promising way to achieve simultaneous steady and instantaneous sensing capabilities, compared to the construction of complex and redundant sensor arrays.

**Figure 1:**
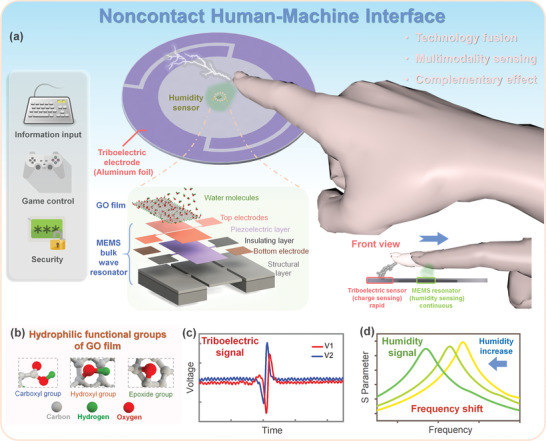
A minimalist and multifunctional noncontact human–machine interface (HMI). a) Schematic illustration of the HMI consisting of a MEMS bulk wave resonant humidity sensor and a triboelectric sensor. b) Structure diagrams of the hydrophilic functional groups of the graphene oxide (GO) film. c) Output voltage waveforms from the triboelectric sensor electrodes when a finger slid over them. d) Resonant frequency shift of the humidity sensor due to the approaching finger.

Herein, by using the complementary sensing capability of the MEMS humidity sensors and the triboelectric sensors, we propose a minimalist and multimodal noncontact interaction interface that can detect steady and instantaneous finger motions simultaneously. The noncontact interaction interface is based on the information fusion of a single MEMS piezoelectric resonant humidity sensor and a facile triboelectric sensor (**Figure** **1**a). With the appropriate structure design, the MEMS humidity sensor and the triboelectric sensor skillfully achieved functional complementarity with each other. The humidity sensor provided a continuous and steady response that the triboelectric sensor cannot achieve, meanwhile, the triboelectric sensor solved the issue that a single humidity sensor cannot recognize multidirectional finger motions. More specifically, the MEMS piezoelectric resonant humidity sensor was based on a 10.5 MHz two‐port bulk wave resonator operating in length extensional mode, rather than well‐investigated surface acoustic wave (SAW) resonators or quartz crystal microbalances (QCMs), to obtain ultratiny form factor, high signal strength, and low signal noise level. Besides, graphene oxide (GO) film rich in various hydrophilic functional groups, e.g., carboxyl, hydroxyl, and epoxide groups (Figure 1b), was selected as the sensing material and coated on the surface of the resonator, to offer the humidity sensor high sensitivity and fast response to ambient humidity changes including the one caused by an approaching finger. Moreover, the high sensing performance of the humidity sensor was highly stable and repeatable because of the uniform and thickness controllable attributes of the prepared GO film. On this basis, the facile triboelectric sensor with an advanced layout and only two electrodes was further integrated with the humidity sensor to achieve functional complementation. The triboelectric sensor was able to rapidly reflect multidirectional finger motions by outputting different voltage waveforms from its two electrodes (Figure 1c), while the humidity sensor could provide a steady and continuous real‐time response to the finger movements through its resonant frequency change (Figure 1d). By leveraging this unique technology fusion, the noncontact interaction interface with minimalist configuration, multimodal sensing capability, and high‐dimensional responses was realized. As a proof‐of‐concept, we demonstrated the applications of this noncontact interaction interface in real‐time information input, virtual car control, and 3D password enter for a login system.

## 2 Humidity Sensor Design and Characterization

**Figure 2:**
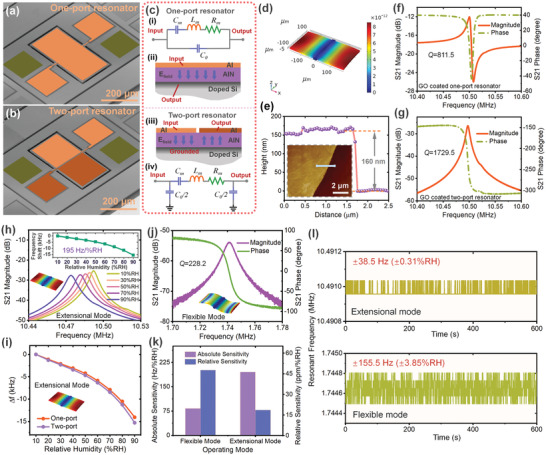
Piezoelectric bulk wave resonant humidity sensor design and characterization. a,b) False‐color SEM images of the prepared piezoelectric bulk wave resonators. c) Electric field distribution and equivalent circuit diagrams of the one‐port and two‐port resonators. d) Simulated length extensional vibration mode of the resonators. e) AFM image and thickness profile of the GO film used. f,g) Transmission spectrums of the one‐port and two‐port resonators coated with GO film. h) Transmission spectrums of the two‐port resonant humidity sensor at different humidity levels. The inserted graph plots the relationship between the sensor's resonant frequency shift and the ambient relative humidity. i) Sensing performance comparison of the one‐port and two‐port resonant humidity sensors. j) Transmission spectrum of the two‐port resonant humidity sensor operating in flexible mode. k) Comparison of the absolute and relative sensitivity of the two‐port resonant humidity sensor operating in different modes. l) Signal fluctuations of the two‐port resonant humidity sensor with different operating modes when the ambient humidity remained constant.

Piezoelectric resonators are the key components that determine the performance of piezoelectric resonant humidity sensors. Compared with other types of piezoelectric resonators, such as SAW resonators, quartz QCMs, and cantilever resonators, bulk wave resonators operating in extensional vibration modes have a more compact size, higher quality factor (*Q*), higher stability, better power handling ability, and lower detection limit, making them more suitable as the sensing platforms.^[^ ^62^, ^63^, ^64^ ^]^ Common piezoelectric bulk wave resonators are mainly composed of a piezoelectric layer, two electrode layers (the piezoelectric layer is sandwiched between the top and bottom electrodes), and a structure layer, as shown in the inset of Figure 1a. Besides, an insulation layer is also used and patterned in the areas of the devices not covered by the piezoelectric layer for electrical isolation of the top electrode. Here the piezoelectric bulk wave resonators were fabricated on a layered structure of an aluminum nitride (AlN) piezoelectric layer and a Si structure layer, with thicknesses of 0.5 and 10 µm. Meanwhile, the surface of the Si layer was highly doped to act as the bottom electrode. The fabrication process is detailed in Figure S1 in the Supporting Information. **Figure** **2**a,b present the SEM pictures (with false colors) of the prepared piezoelectric bulk wave resonators with different top electrode designs, named one‐port resonator and two‐port resonator. Both resonators had the same plane size of 200 × 400 µm^2^. Different from the single top electrode design of the one‐port resonator, the top electrode of the two‐port resonator was divided into two parts, to reduce the influence of the feedthrough capacitance between resonator signal input and output terminals on the sensor signal strength.^[^ ^65^ ^]^ The specific mechanism can be illustrated by equivalent circuit diagrams and the electric field (E_field_) distribution of the resonators shown in Figure 2c. Figure 2ci gives the equivalent circuit of the one‐port resonator, which describes the electrical response of the vibrating resonator in the test electronic circuit. The resistance (*R*_m_), inductance (*L*_m_) and capacitance (*C*_m_) in the equivalent circuit are associated with the damping, inertia and compliance of the resonator in the mechanical system, respectively. In addition, the capacitance (*C*_0_) represents the intrinsic capacitance (feedthrough capacitance) between the signal input and output terminals, which largely attenuates the signal strength of the resonator. For the one‐port resonator, the value of *C*_0_ is relatively large, because the top and bottom electrodes arranged in parallel act as the signal input and output terminals, as indicated in Figure 2cii. While for the two‐port resonator (Figure 2ciii), the separated two top electrode parts serve as two signal terminals, and the bottom electrode is grouped. Benefit by the electrical isolation of the signal input and output terminals, the feedthrough capacitance can be effectively reduced, which can be reflected in the equivalent circuit of two‐port resonator in Figure 2civ.

Excited by an external signal, both resonators could generate extensional vibrations along their length direction, as indicated by the COMSOL simulation results shown in Figure 2d. Apart from the sensing platform, humidity sensing materials also play an important role in the performance of humidity sensors. GO film as a carbon nanomaterial, in addition to having a large surface‐to‐volume ratio, is also rich in various hydrophilic functional groups and able to rapidly absorb and desorb water molecules, making it a promising sensing material for humidity sensors.^[^ ^66^, ^67^, ^68^ ^]^ To achieve a reproducible performance of the humidity sensors, vacuum filtration was adopted to prepare the GO film with uniform and controllable thickness, in which anodic aluminum oxide (AAO) membrane with large porosity was used as the filter membrane (Figure S2a, Supporting Information). The prepared uniform GO film attached to the AAO membrane is shown in Figure S2b in the Supporting Information. By leveraging the surface tension of the DI water, the GO film was separated from the AAO membrane spontaneously and then transferred to the surface of the resonators.^[^ ^69^ ^]^ Figure 2e displays the AFM image and thickness profile of the GO film (around 160 nm) used in this work. With the GO film overlaid on the surface, the one‐port and two‐port resonators were tested for resonant frequency response in the atmospheric environment with a network analyzer (R&S ZNB20). Comparing the test results plotted in Figure 2f,g, it can be observed that benefited from the divided top electrode design, the signal strength of the two‐port resonator (*Q* = 1729.5) at the resonant frequency of 10.5 MHz was distinctly higher than that of the one‐port resonator (*Q* = 811.5), which was beneficial to improve the stability of the humidity sensor during the test.

A dedicated test setup was then built to perform humidity response characterization of the humidity sensors (Figure S3, Supporting Information), which could adjust the humidity level in the test environment from 0.1% to 95%RH with an adjustment accuracy of 0.1%RH. Figure 2h provides the transmission spectrums of the two‐port resonant humidity sensor at different humidity levels. With the increment of ambient humidity, the GO film covered on the resonator surface gradually adsorbed water molecules, increasing its mass, which caused the resonant frequency of the resonator to shift to low frequencies. These test results (insert graph in Figure 2h) also revealed that the humidity sensor possessed a high average sensitivity of 195 Hz %RH^–1^ when the humidity changed from 10% to 90%RH. Additionally, no significant signal strength attenuation was observed throughout the test, and the high humidity sensing performance of the sensor was highly repeatable (Figure S4a, Supporting Information). Furthermore, the high stability and good power handling capability of the bulk wave resonator ensured the humidity sensor with unaltered response performance under different driving powers, as evident in Figure S4b in the Supporting Information. Besides, what is also worth noting is that the humidity sensor showed low humidity hysteresis (less than 3%RH) during the desorption process (Figure S4c, Supporting Information). Compared with the response of the one‐port resonant humidity sensor shown in Figure 2i, the two‐port resonant humidity sensor had a slightly higher sensitivity, which was mainly because of the contribution from GO film conductivity changes during the test.

In addition to the length extensional mode, the flexible mode was another type of vibration mode that can be excited in the two‐port resonant humidity sensor as depicted in Figure 2j. Same humidity response characterization was then conducted for the two‐port resonant humidity sensor operating in its flexible mode, and the test results are provided in Figure S5, Supporting Information. Figure 2k comprehensively compares the absolute sensitivity and relative sensitivity (ratio of absolute sensitivity to resonant frequency) of the two‐port resonant humidity sensor operating in length extensional mode and flexible mode, respectively. Due to the lower resonant frequency (around 1.74 MHz), the resonator humidity sensor operating in flexible mode possessed much lower absolute sensitivity than that in length extensional mode, whereas it had an apparent advantage in relative sensitivity because the out‐of‐plane vibration modes (flexible mode is one of the out‐of‐plane vibration modes) are more sensitive to mass changes than the in‐plane vibration modes (length extensional mode is one of the in‐plane vibration modes).^[^ ^70^, ^71^ ^]^ Nevertheless, the low *Q* factor (*Q* = 228.2) of the flexible mode affected the stability of the test signal and increased the signal noise level, which is verified by the test results in Figure 2l. When the ambient humidity remained constant, the signal noise value of the humidity sensor operating in the flexible mode reached nearly ±155.5 Hz (equivalent to ±3.85%RH according to its humidity sensitivity), while the noise value of the sensor operating in length extensional mode was only ±38.5 Hz (±0.31%RH). Taking the above test results into consideration, the two‐port resonant humidity sensor operating in length extensional mode, with high signal strength and low signal noise level, was the best choice for constructing noncontact HMIs, and more characterization tests were subsequently carried out on it.

## 3 Dynamic Response of the Two‐Port Resonant Humidity Sensor

**Figure 3:**
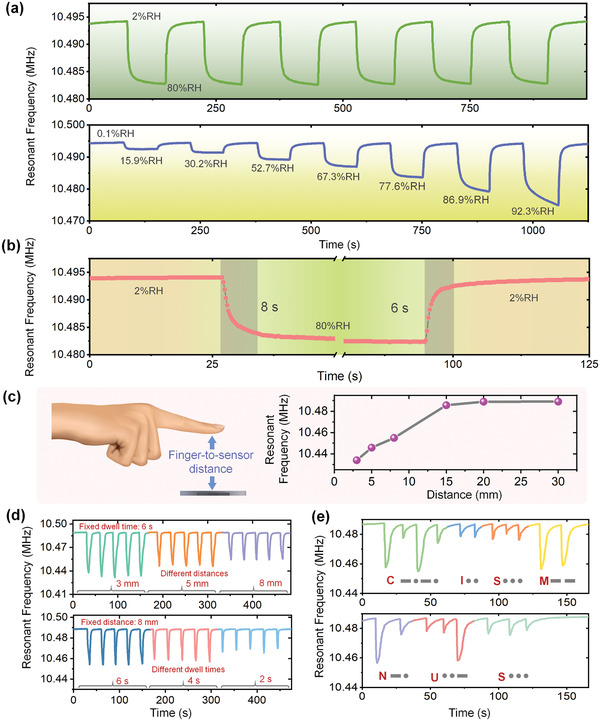
Dynamic response of the two‐port resonant humidity sensor and its response to a repeated approaching finger. a) Continuous response of the humidity sensor when humidity varied within the same and different ranges. b) Detailed response and recovery processes of the humidity sensor. c) Relationship between the humidity sensor resonant frequency and the finger‐to‐sensor distance. d) Response of the humidity sensor to a repeated approaching finger. e) Noncontact input words of “CISM NUS” in the form of Morse code.

The dynamic response of the two‐port resonant humidity sensor operating in length extensional mode was then investigated by continuously changing the ambient humidity. **Figure** **3**a provides the recorded real‐time resonant frequency change of the humidity sensor when the ambient humidity was changed in the same and different ranges. As evident from these test results, the humidity sensor possessed excellent repeatability and stability over a wide humidity test range, even at humidity levels less than 1%RH and greater than 90%RH. Additionally, Figure 3b plots the detailed response and recovery processes of the sensor after quickly switching the ambient humidity between 2% and 80%RH, and the overall required response and recovery time were only 8 seconds and 6 seconds, respectively.

For noncontact human–machine interaction, the actual response of the humidity sensors to the humidity changes brought about by the proximity of human fingers is crucial. Figure 3c shows the relationship between the humidity sensor's resonant frequency and the finger‐to‐sensor distance. When the finger was far away (greater than 20 mm), the humidity sensor had almost no response, and when the finger‐to‐sensor distance was shortened, the high humidity on the finger surface gradually affected the sensor, resulting in a decrease in the sensor's resonant frequency. Since the noncontact human–machine interaction is a dynamic process in practical applications, the dynamic response performance of the humidity sensor to the approaching of a human finger was further investigated. The humidity sensor was fixed on a panel during the test and its response was recorded in real‐time using a laptop with the LabVIEW program connected to the VNA (Video S1, Supporting Information). As shown in Figure 3d, both the finger‐to‐sensor distance and the finger dwell time affected the response of the humidity sensor to various degrees, because of the different resulting humidity changes around the sensor. The closer the distance and the longer the dwell time, the larger the resonant frequency shift of the sensor, however a dwell time of one to two seconds was enough for the sensor to produce a noticeable response. Therefore, specific sensor responses could be controlled artificially by changing the distance and dwell time of the finger, which provided the possibility to realize noncontact information input. Figure 3e plots the information “CISM NUS” in the form of Morse code entered by contactless means (Video S2, Supporting Information), where the large resonant frequency shift (resonant frequency less than 10.47 MHz) of the sensor represented “dah”, and the small frequency shift represented “dit” (resonant frequency between 10.48 and 10.47 MHz).

## 4 Triboelectric Sensor Design and Characterization

Through the GO‐coated MEMS piezoelectric bulk wave resonator, humidity sensing and noncontact finger interaction were achieved for various monitoring and remote‐control applications. Yet due to the demand for high‐dimensional control, while keeping the interaction interface system lean, a complementary noncontact method was desired to be integrated for more practical and real‐time functionality. Herein, we designed a facile triboelectric sensor with minimalist two annular electrodes and the same noncontact characteristics to enable multidirectional finger motion sensing. On one hand, the triboelectric sensor was able to capture the remote finger motions instantly, facilitating the rapid detection of multidirectional and dynamic finger interactions. On the other hand, the humidity sensor with a steady monitoring ability could be used for detecting the continuous operations of the finger, which was difficult to be achieved only with triboelectric sensors. Thus, with an integrated triboelectric sensor, the resultant interface was able to achieve not only steady and dynamic motion sensing, but also higher‐dimensional signals for more complex controls and interactions.

**Figure 4:**
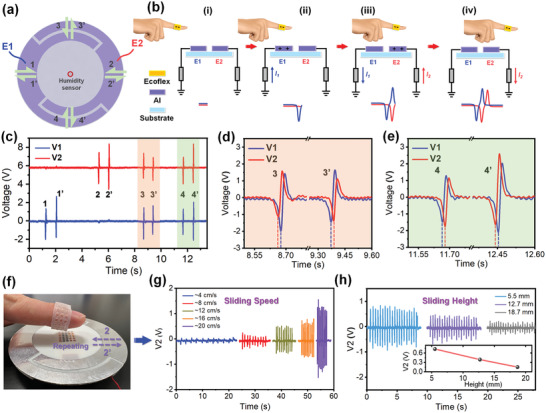
Noncontact minimalist triboelectric sensor design and characterization. a) Designed electrode pattern of E1 and E2 for multidirectional finger motion sensing. b) Operation principle of charge flow when the finger slid over the electrode pattern in direction 4. c) Distinguishable output waveforms from different sliding directions. d) Detailed output waveforms for the sliding direction of 3/3'. e) Detailed output waveforms for the sliding direction of 4/4'. f) Photograph and indication of the repeated finger sliding in the direction of 2/2' for the later characterization. g) Influence of the finger sliding speed on the outputs. h) Influence of the finger sliding height on the outputs. The inserted graph gives the relationship between the output voltage amplitude of the triboelectric sensor and the finger sliding height.

The proposed triboelectric sensor was constructed with a facile configuration, consisting of only a patterned electrode layer and an interactive Ecoflex ring attached to the fingertip (the Ecoflex ring was only needed in high humidity areas like Singapore). The layout of the patterned electrode is shown in **Figure** **4**a, which can be conveniently fabricated on both rigid and flexible substrates according to the corresponding applications. Two electrodes, i.e., E1 and E2, were patterned around the humidity sensor, forming the particular and minimalist pattern for 4‐directional interactions. The directions of the remote sliding motions (in/out) were defined as 1/1', 2/2', 3/3', and 4/4', indicated by the arrows in Figure 4a. With the outputs from direction 4 as an example, the basic operation principle of the noncontact triboelectric sensing is illustrated in Figure 4b. In Figure 4bi, after the initial contact with common materials such as human skin, clothes, etc., the Ecoflex ring on the fingertip contained electrons on its surface due to its high electronegativity. It is worth noting that air‐permeable holes (with a diameter of ≈1 mm) were created on the Ecoflex ring, in order to achieve minimal influence on the finger humidity for the humidity‐actuated interactions. When the finger slid on top of E1 (Figure 4bii), electrons on E1 were repelled to the ground due to the electrostatic induction, thus generating a negative output current on the connected circuit. As the finger continuously slid away from E1 and on top of E2 (Figure 4biii), electrons flowed back to E1 to maintain electrostatic equilibrium, generating a positive output current. Meanwhile, E2 underwent a similar change as E1 in the previous state, producing a negative current on the connected circuit. Last, when the finger slid away from E2, a positive output current was then generated (Figure 4biv).

Generally speaking, when the finger slid across single‐electrode patterns (direction 1/1' and 2/2“), outputs were only generated on the respective electrode. If the finger slid across the double‐electrode patterns (direction 3/3” and 4/4“), outputs were generated on both electrodes, with the signal sequence depending on the sliding sequence. Therefore, with different sliding directions, distinguishable output waveforms were produced on E1 and E2 instantly, as depicted in Figure 4c (Video S3, Supporting Information). Figure 4d,e indicate the detailed output waveforms for directions 3/3” and 4/4' with a zoomed‐in view. These distinguishable waveforms were adopted to differentiate multidirectional fingertip interactions. A real photograph of the fabricated triboelectric sensor and the Ecoflex ring is shown in Figure 4f. Next, the influencing factors on the outputs were also investigated. Figure 4g shows the effect of sliding speed on the outputs, indicating that a higher speed generated a larger output signal. Then as in Figure 4h, it was found out that a lower sliding height produced a larger output signal, which is consistent with the electrostatic induction theory (the inserted graph in Figure 4h gives an intuitive view of the relationship between the triboelectric sensor's output voltage amplitude and the finger sliding height). With a normal sliding speed and sliding height (≈1 cm), adequate output amplitude and signal‐to‐noise ratio could be achieved for later signal processing and interactive applications.

## 5 Noncontact Game Control Interface

With the complementary characteristics and functionalities, the MEMS humidity sensor and triboelectric sensor were integrated with data acquisition modules, a microcontroller unit and a laptop, forming a multimodal sensory and control system for various practical applications. Enabled by the technology fusion of MEMS and TENG, the integrated interaction interface was able to contactless capture multimodal sensory information, including both the dynamic finger motion and the steady finger humidity, for rapid and continuous interactions respectively.

**Figure 5:**
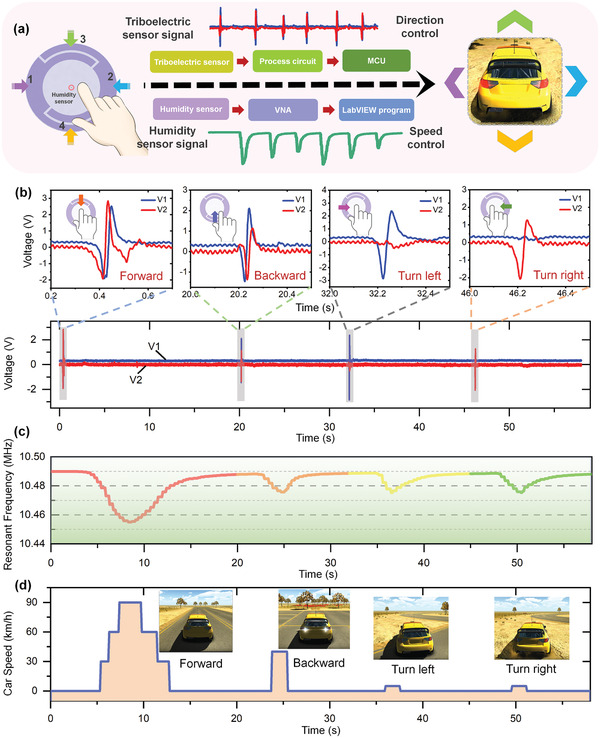
Noncontact car control using the multimodal sensory outputs of the integrated system. a) Schematic diagram of the output flow for car direction and speed control. b) Two‐channel triboelectric voltages generated from the finger sliding motions. c) Resonant frequency of the MEMS humidity sensor throughout the finger operation. d) Corresponding car direction and speed control during the noncontact finger‐interactive process.

To show the actual capability of the integrated system, car control in virtual reality (VR) space was demonstrated using defined finger interactions. As indicated in **Figure** **5**a, finger motion‐induced triboelectric outputs were captured by an acquisition circuit and then sent to a microcontroller unit for signal detection and processing. At the same time, finger humidity‐induced resonance outputs were captured by the VNA and read by the LabVIEW program to record the resonant frequency. Afterward, both detected signals from the finger motion and finger humidity were sent to a Unity3D program on a laptop, to control the moving direction and speed of the car.

The generated output signals from finger interactions are plotted in Figure 5b,c, indicating the respective two‐channel triboelectric voltages and MEMS humidity sensor's resonant frequency. Meanwhile, the graph and insets in Figure 5d show the corresponding controlled car moving directions and speeds in the VR space. In a typical interactive process, a finger wearing the Ecoflex ring first slid over the triboelectric electrode pattern along direction 3, output voltages were then generated on E2 and E1 consecutively (Figure 5b). This particular output voltage pattern was detected by the microcontroller unit, which then sent a control command to Unity3D to enable the car in a forward standby mode. During this process, the resonant frequency of the MEMS humidity sensor was maintained since the finger was far away from it. After the sliding motion, the finger held its position over the MEMS humidity sensor, inducing a gradual decrement of the resonant frequency of the humidity sensor (Figure 5c). The humidity level and the humidity sensor's resonant frequency were controlled by the finger‐to‐sensor distance and finger dwell time. As the resonant frequency decreased beyond a certain defined value, the car started to move in the forward direction. Thanks to the steady change of the humidity, continuous speed control of the car was possible based on the humidity sensor's resonant frequency, but for easy interaction, 4 speeds were defined here for the forward mode: 0 km h^−1^ (resonant frequency > 10.48 MHz), 30 km h^−1^ (10.47 MHz < resonant frequency ≤ 10.48 MHz), 60 km h^−1^ (10.46 MHz < resonant frequency ≤ 10.47 MHz), and 90 km h^−1^ (resonant frequency ≤10.46 MHz). The moving speed variation and car moving screenshots can be observed in Figure 5d. Then with the finger gradually moving away from the MEMS humidity sensor, the car moving speed decreased accordingly and eventually becomes zero when the resonant frequency was above the defined value. Following the forward control, the finger slid along directions 4, 1, and 2 consecutively and held over the MEMS humidity sensor in a similar manner, for controlling the car to move back, turn left, and turn right, respectively. What is worth mentioning is that for better visualization, only a two‐speed level was set for the last 3 modes. That is, when the resonant frequency of the humidity sensor was lower than 10.48 MHz, the corresponding moving speeds of the car backward, left turn and right turn were 40, 5, and 5 km h^−1^, respectively, and when the resonant frequency was higher than 10.48 MHz, the speed of the car returned to 0 km h^−1^. The demonstration of car control can be found in Video S4 in the Supporting Information, which shows that the car moving direction and speed were controlled conveniently with the noncontact, multimodal, and complementary sensory information from the integrated interaction interface.

## 6 Noncontact 3D Password Input Interface

**Figure 6:**
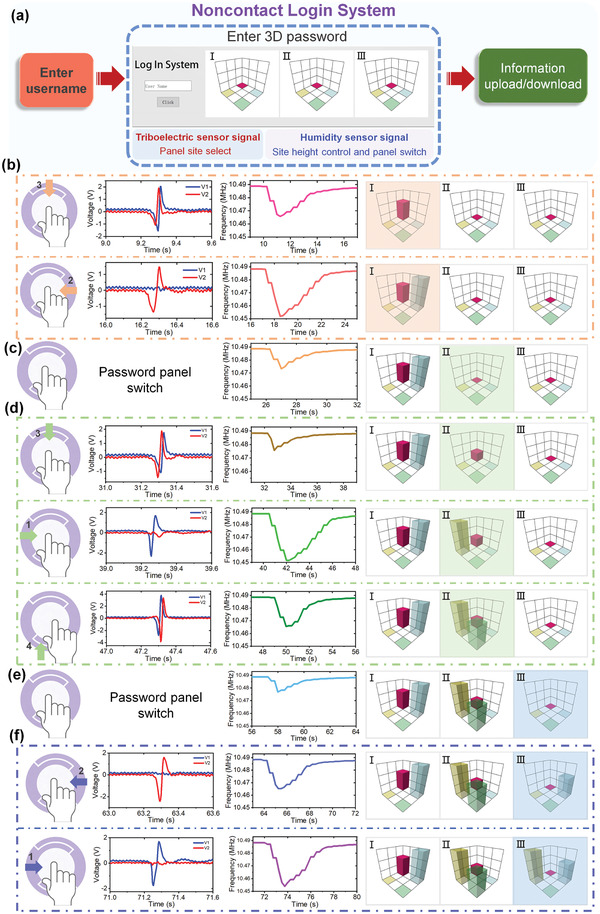
Noncontact 3D password interface for a login system. a) Schematic illustration of the noncontact login system. b) Site selection and height control of password panel I based on the triboelectric sensor and humidity sensor signals. c) Switch from password panel I to password panel II based on the humidity sensor signal. d) Site selection and height control of password panel II based on the triboelectric sensor and humidity sensor signals. e) Switch from password panel II to password panel III based on the humidity sensor signal. f) Site selection and height control of password panel III based on the triboelectric sensor and humidity sensor signals.

Except for the gaming control application, the proposed interaction interface can also realize noncontact 3D password input in a login system. **Figure** **6**a shows the schematic diagram of the noncontact login system. Each user was able to log into the system by entering his username and unique 3D password on the password input interface, and then perform operations within the limits of his authority. The password input interface consisted of three independent password display panels, each of which had four control sites corresponding to four orientations of the triboelectric sensor on the noncontact interaction interface, and the height of the sites was controlled by the signal of the humidity sensor to realize 3D password input (humidity sensor resonant frequency between 10.47 and 10.48 MHz, between 10.46 and 10.47 MHz, and less than 10.46 MHz yielded low, medium and high site heights, respectively). Meanwhile, the switching between the password panels was also completed by the humidity sensor signal, and the number of the password panels as well as the height levels can be increased according to the needs of the encryption level. Figure 6b–f describes in detail the process of noncontact input 3D passwords on password panels I, II, and III in sequence. As shown in Figure 6b, the finger first slid over the triboelectric sensor along direction 3 (as defined in Figure 4a), and the top site of panel I was activated according to the triboelectric sensor signal. Immediately after, the finger moved close to the humidity sensor to shift the resonant frequency of the sensor to between 10.46 and 10.47 MHz, a medium height rectangular column was then generated at this site. Whereafter, the finger slid along direction 2 over the triboelectric sensor and stopped near the humidity sensor, to activate the right site of the panel I and create a high height rectangular column. After completing the password input of panel I, the finger skipped sliding the triboelectric sensor and directly approached the humidity sensor, and shifted the resonant frequency of the sensor to between 10.47 and 10.48 MHz to switch from panel I to panel II, as revealed in Figure 6c. Similar operation processes were carried out to accomplish the password input on panels II and III, and the switch from panel II to panel III (Figure 6d–f). With the correct 3D password related to the usernames, the users could successfully log into the system and complete their demanded operations according to their authorities set in the system, e.g., uploader or reader (Video S5, Supporting Information). An uploader could upload and read a file in the cloud database, while a reader only had the access to read the file. In addition to providing a new and highly secured 3D password, this noncontact input interface also effectively solved the shortcomings of traditional contact password input systems, which are easy to spread viruses and have low security due to residual fingerprints.

## 7 Conclusions

In summary, we designed and integrated a humidity sensor and a triboelectric sensor to develop a minimalist, multimodal noncontact interaction interface for various multidimensional control applications. The humidity sensor was designed to recognize continuous and steady command signals during noncontact interactions, while the triboelectric sensor was intended for the identification of fast and instantaneous signals. With the advanced two‐port resonant design and uniform GO film coating, the MEMS humidity sensor successfully achieved high sensitivity, low signal noise, excellent repeatability, and rapid response and recovery speed over a wide humidity range. Benefited by these characteristics, the humidity sensor can be applied for monitoring continuous finger interactions based on different finger‐to‐sensor distances and finger dwell time. On this basis, the triboelectric sensor with a facile two‐electrode design was then integrated with the humidity sensor to realize the complementary monitoring of multidirectional and dynamic finger movements. With this unique complementary information fusion, the proposed noncontact interaction interface was able to provide multimodal continuous and dynamic sensory information for various human–machine interactions with lower power consumption and simpler system structure, as compared with other noncontact HMIs (Table S1, Supporting Information). To demonstrate its feasibility in practical applications, the interaction interface was successfully applied for VR car control and 3D password input by using intuitive finger interactions. Moving forward, the applications of this noncontact interaction interface can be extended to more wearable fields in the Metaverse with a flexible or textile substrate as the supporting platform.

## 8 Experimental Section

### Fabrication of the Piezoelectric Bulk Wave Resonators

The fabrication of the piezoelectric resonators started with an n‐type silicon‐on‐insulator (SOI) wafer (Figure S1, Supporting Information), and the surface of the wafter was first deposited with a layer of phosphosilicate glass (PSG). After that, the wafter together with the PSG layer was annealed at 1050 °C for about an hour to realize a highly doped Si surface, which was later used as the bottom electrode. The PSG layer was then removed by wet chemical etching. Subsequently, a 200 nm thermal oxide was grown on the Si surface and patterned by reactive ion etching (RIE) for electrical insulation of the device. Next, a 0.5 µm AlN layer was deposited and wet etched to act as the piezoelectric layer. Whereafter, a metal stack of 20 nm Cr and 1 µm was consecutively deposited and patterned through a lift‐off process to form the top electrodes and wire bonding pads. Later on, the Si structure layer was pattered by deep reactive ion etching (DRIE) to define the device structure. Lastly, the Si substrate layer and SOI oxide layer were patterned by DRIE and wet etching, respectively, to release the device.

### Preparation and Transfer of the Uniform GO Film

A 0.4 wt% GO water dispersion (purchased from Graphenea, Inc.) was diluted to 0.02 wt% using deionized (DI) water. Then, 1 mL of the diluted GO dispersion was transferred to the Buchner funnel of the vacuum filtration set up with a pipette. The Buchner funnel was pre‐filled with 150 mL of DI water, and an anodic aluminum oxide (AAO) membrane (Whatman 6809‐5002) was placed below as a filter membrane. After the water in the Buchner funnel was vacuumed out, a uniform GO film attached to the AAO film was obtained. Subsequently, the GO film together with the AAO membrane was placed on the surface of DI water. Under the action of the water surface tension, the GO film gradually separated from the AAO membrane and continued to float on the water surface. Finally, the piezoelectric resonator was immersed in the DI water and lifted up from the bottom of the GO film to complete the GO film transfer.

### Characterization of the Humidity Sensor

During the humidity response characterization, the humidity sensor was fixed in a metallic chamber (Figure S3, Supporting Information). The humidity level inside the chamber was manually adjusted between 0.1% and 95%RH by changing the dry and wet N_2_ flow rates. Meanwhile, the outlet of the chamber was connected to a commercial hygrothermograph (AZ8721) to calibrate the humidity and temperature inside the chamber. The humidity response of the sensor was detected and recorded by a network analyzer (R&S ZNB20) and a PC with an installed LabVIEW program.

### Preparation of the Ecoflex Ring and Triboelectric Sensor

For the preparation of the Ecoflex ring, the Ecoflex 00‐30 solution (mass ratio of Part A and Part B is 1:1) was poured into a 3D printed mold, solidified to form a belt, and then taken out and fixed into a ring shape with glue (Figure S6a, Supporting Information). An air‐permeable hole array was patterned on the Ecoflex ring to minimize the influence on the finger humidity for the humidity‐actuated interactions (Figure S6b, Supporting Information). Besides, an aluminum tape was cut into the electrode shape required by the triboelectric sensor and attached to a panel, the humidity sensor was embedded in the center of the panel (Figure S6c, Supporting Information). During the actual operation, the finger with the Ecoflex ring slid above the interaction interface without contact (Figure S6d, Supporting Information). It is worth mentioning here that, according to the actual application requirements, the humidity sensor and triboelectric sensor were able to be integrated on a flexible substrate to form a flexible and wearable noncontact interaction interface, as shown in Figure S7 in the Supporting Information.

### Characterization and Signal Recognition of the Triboelectric Sensor

The output voltages from the triboelectric sensor electrodes were measured by an oscilloscope (Agilent DSO‐X3034A) to characterize the sensor's noncontact interaction performance. While during the application demonstrations, the output voltage waveforms were recognized by a programmable Arduino UNO and converted into corresponding control commands

## Conflict of Interest

The authors declare no conflict of interest.

## Supporting information

Supporting Information

Supplemental Video 1

Supplemental Video 2

Supplemental Video 3

Supplemental Video 4

Supplemental Video 5

## Data Availability

The data that support the findings of this study are available in the supplementary material of this article.
