# Supplementary material for: Noncontact Human–Machine Interface Using Complementary Information Fusion Based on MEMS and Triboelectric Sensors
Source: Adv Sci (Weinh). 2022 May 18;9(21):2201056. doi: 10.1002/advs.202201056 (PMC9313506; doi:10.1002/advs.202201056)
Supplement: Supplementary file 1 — Supporting Information [file ADVS-9-2201056-s001.pdf]

Supporting Information

Noncontact Human-Machine Interface Using Complementary Information Fusion Based on MEMS and Triboelectric Sensors

*Xianhao Le[1], Qiongfeng Shi[1], Zhongda Sun, Jin Xie, and Chengkuo Lee**

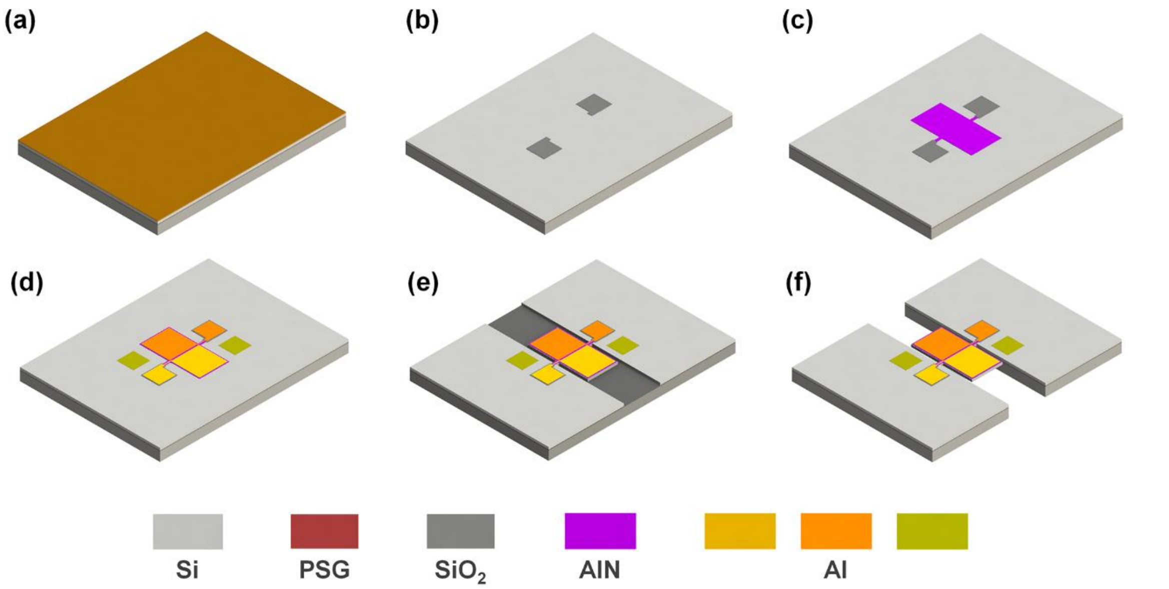

Figure S1. Fabrication process of the bulk wave resonator. a) A phosphosilicate glass (PSG) layer was deposited on an n-type SOI wafer and annealed to achieve Si surface highly doping, and then removed by wet chemical etching. b) A 200 nm thermal oxide layer was grown and patterned. c) A 0.5 μm AlN layer was deposited and wet etched. d) A metal stack (20 nm Cr and 1 μm Al) was consecutively deposited and patterned through a lift-off process. e) The Si structure layer was patterned by deep reactive ion etching (DRIE). f) The Si substrate layer and oxide layer were patterned by DRIE and wet etching, respectively.

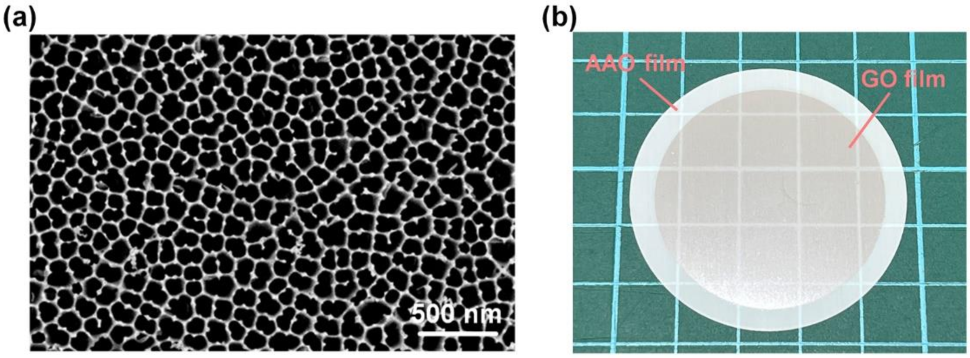

Figure S2. Structures of AAO film and prepared GO film. a) SEM image of the AAO film with large porosity. b) Optical picture of the prepared GO film attached to the AAO film.

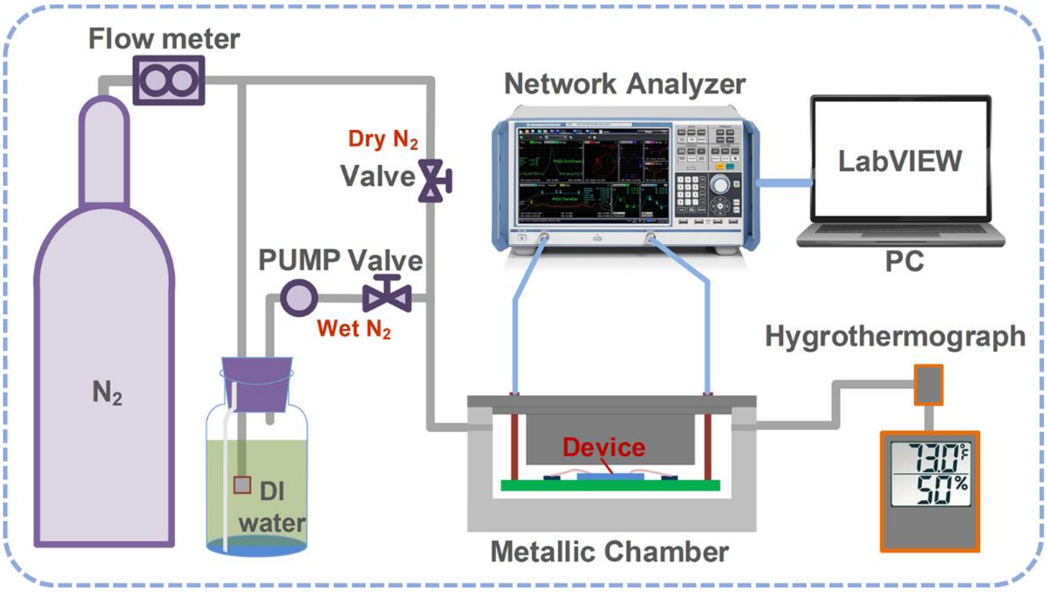

Figure S3. Experiment setup for humidity sensor characterization.

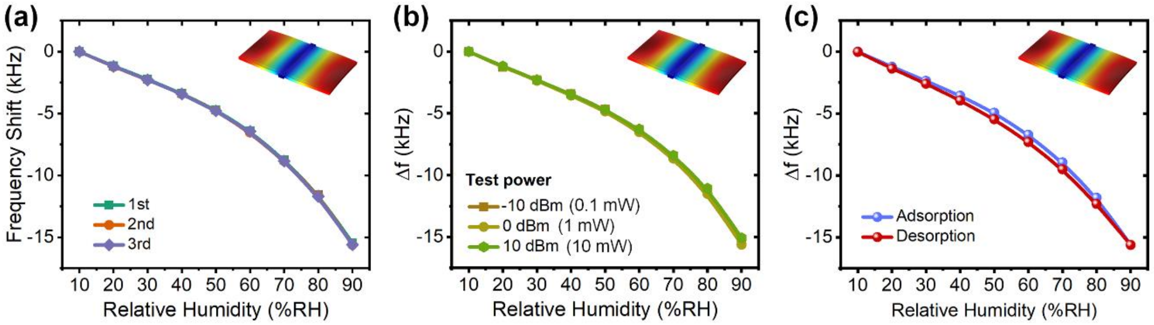

Figure S4. Humidity response of the two-port resonant humidity sensor operating in length extensional mode. a) The relationship between the sensor resonant frequency shift and ambient relative humidity under repeated tests. b) Sensing performance of the sensor with different driving power. c) Humidity hysteresis characterization of the sensor during the desorption process.

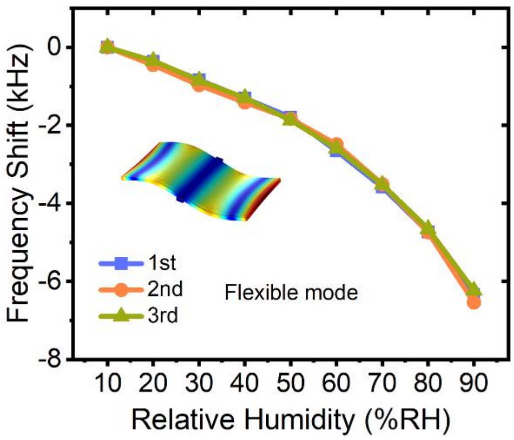

Figure S5. Humidity response of the two-port resonant humidity sensor operating in flexible mode.

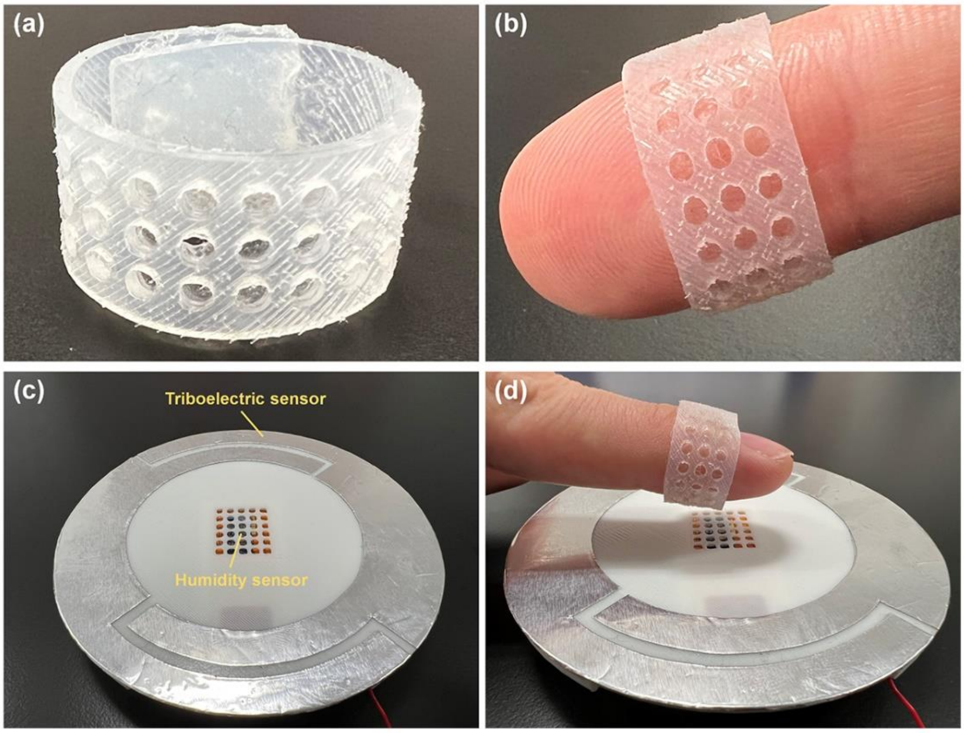

Figure S6. Photographs of the Ecoflex ring and the integrated device. a) Fabricated Ecoflex ring using a 3D printed mold. b) The Ecoflex ring worn on a finger. c) The integrated device with MEMS humidity sensor in the center and triboelectric electrodes at the edge. d) The finger holding over the interacting interface.

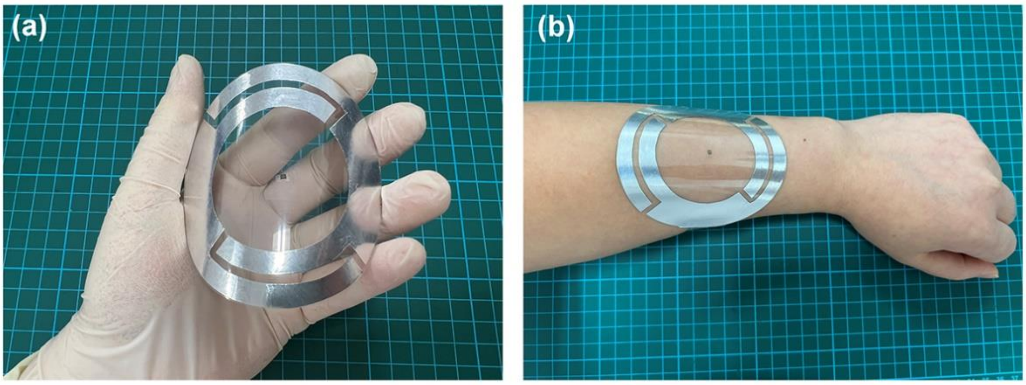

Figure S7. Flexible and wearable version of the integrated device with MEMS humidity sensor in the center.

Table S1. Summary of recent noncontact HMI systems.

Ref.	Sensors	System Structure	Dynamic Response Speed	Steady Response	Power Consumption
[1]	Ultrasonic sensor array	Complex	Fast	Yes	High
[2]	Optical sensor array	Complex	Fast	Yes	High
[3]	Piezoelectric micromachined ultrasound transducers	Complex	Fast	Yes	Low
[4]	Triboelectric sensor array	Complex	Ultra-fast	No	Self-powered
[5]	Humidity sensor array	Complex	Fast	Yes	Low
This work	Integration of a humidity sensor and a triboelectric sensor	Simple	Ultra-fast	Yes	Ultra-low

Supplementary References

[1] F. Zhou, X. Li, Z. Wang, *IEEE Sens. J.* **2020**, *20*, 13501.

[2] K. Czuszynski, J. Ruminski, A. Kwasniewska, *IEEE Sens. J.* **2018**, *18*, 5429.

[3] P. Gijsenbergh, A. Halbach, Y. Jeong, G. B. Torri, M. Billen, L. Demi, C. H. Huang, D. Cheyns, X. Rottenberg, V. Rochus, *J. Micromechanics Microengineering* **2019**, 29, 074001.

[4] Y. Tang, H. Zhou, X. Sun, N. Diao, J. Wang, B. Zhang, C. Qin, E. Liang, Y. Mao, *Adv. Funct. Mater.* **2020**, 30, 1907893.

[5] L. Lu, C. Jiang, G. Hu, J. Liu, B. Yang, *Adv. Mater.* **2021**, 33, 2100218.

Supporting Videos

Video S1. Noncontact sensing_humidity sensor.

Video S2. Noncontact information input.

Video S3. Noncontact sensing_triboelectric sensor.

Video S4. Noncontact car game control.

Video S5. Noncontact 3D password input.
